# Comparison of percutaneous endoscopic interlaminar discectomy and conventional open lumbar discectomy for L4/5 and L5/S1 double-segmental lumbar disk herniation

**DOI:** 10.1186/s13018-023-04361-9

**Published:** 2023-12-11

**Authors:** Yingchuang Tang, Hanwen Li, Wanjin Qin, Zixiang Liu, Hao Liu, Junxin Zhang, Haiqing Mao, Kai Zhang, Kangwu Chen

**Affiliations:** 1 Department of Orthopaedic Surgery, The First Affiliated Hospital of Soochow University, 899 Pinghai Road, Suzhou, 215000 Jiangsu China; 2 Department of Orthopaedic Surgery, Wujin Hospital Affiliated With Jiangsu University, Changzhou, Jiangsu China

**Keywords:** Conventional open lumbar discectomy, Percutaneous endoscopic interlaminar discectomy, Double-segmental lumbar disk herniation, Spinal endoscopy

## Abstract

**Objective:**

Although spinal endoscopic techniques have shown great advantages in the treatment of single-segment lumbar disk herniation (LDH), the therapeutic advantages for double-segment LDH are controversial. To compare the outcomes of percutaneous endoscopic interlaminar discectomy (PEID) versus conventional open lumbar discectomy (COLD) for the treatment of L4/5 and L5/S1 double-segmental LDH.

**Methods:**

From January 2016 to September 2021, we included 50 patients with double-segmental LDH who underwent PEID (*n* = 25) or COLD (*n* = 25). The clinical outcomes between the two groups were evaluated using the visual analog scale (VAS), the Oswestry disability index (ODI), and the modified MacNab criteria. Moreover, the incision length, operation time, intraoperative fluoroscopy time, postoperative bedtime, hospital stays, and complications were also recorded and compared after surgery.

**Results:**

In both groups, the VAS and ODI scores at different timepoints postoperatively were significantly improved compared with those preoperatively (*P* < 0.05) According to the modified MacNab criteria, the excellent or good outcome rate was 92% in the PEID group and 88% in the COLD group. The PEID group had shorter incision length, postoperative bedtime, and hospital stays than the COLD group. However, the operation time was shorter and intraoperative fluoroscopy time was fewer in the COLD group. In addition, there was no significant difference between the two groups in terms of surgical complications during the postoperative follow-up period.

**Conclusions:**

Both PEID and COLD have good efficacy and high safety for management of L4/5 and L5/S1 double-segmental LDH. Compared with the COLD group, the PEID group had more operative time as well as more intraoperative fluoroscopy, but it had a more minimally invasive surgical incision as well as faster postoperative recovery.

## Introduction

Lumbar disk herniation (LDH) is one of the most common degenerative diseases of the lumbar spine, with > 95% occurring at the L4/5 or L5/S1 level [1]. LDH is often accompanied by nerve compression symptoms such as sciatica, leg pain, or lower back pain. In clinic, we found that it is not rare for patients to develop double-level LDH, while patients who fail to receive stepwise conservative treatment always need further surgical interventions [2]. Conventional open lumbar discectomy (COLD) and percutaneous endoscopic lumbar discectomy (PELD) are the common surgical approaches for LDH [3].

The surgical approach for LDH treatment has dramatically improved recently. The first discectomy was performed by Krause in 1908 [4], which remains the standard procedure for LDH to date [5, 6]. The first microdiscectomy was performed by Yasargil in 1977, showing the advantages of simplicity of operation, fewer complications, and satisfying outcomes [7, 8]. With the development of endoscopy and instrumentation, lumbar discectomy (PELD) has increasingly gained attention for the treatment of LDH due to its advantages of less bleeding and trauma, faster recovery, and stable effectiveness. Percutaneous endoscopic interlaminar discectomy (PEID), a PELD procedure, has the advantage of avoiding iliac crest obstruction, quicker puncture positioning, shorter operative time, and less intraoperative radiation exposure via the interlaminar approach. It is considered to be particularly suitable by spine surgeons for the treatment of L5/S1 disk herniations [9, 10]. Due to prolonged sedentary behaviors and lack of exercise, LDH is increasingly observed in young people and the number of patients with double-segmental LDH is increasing. However, only a few studies have investigated the outcomes of PEID versus COLD for LDH at both L4/L5 and L5/S1 segments. The choice of surgery for patients with double-segment LDH is still debatable [3]. Therefore, we conducted a comparative study using a cohort of patients with double-segmental LDH who underwent COLD or PEID. The aims of this study were as follows: (1) to evaluate the safety and efficacy of PEID and COLD in the treatment of double-segmental LDH and (2) to compare the clinical outcomes and postoperative complications based on the two techniques.

## Methods and materials

### Patient population

This study was approved by the Ethical Committee of the First Affiliated Hospital of Soochow University. Informed consent was obtained from all patients. In this retrospective study, from January 2016 to September 2021, we included 50 patients with double-segmental LDH who underwent PEID (*n* = 25) or COLD (*n* = 25). Basic demographic information including age, sex, mean follow-up time, body mass index, smoking rate, symptoms, and physical signs was collected. For patients with L4/5 and L5/S1 double-segmental LDH, they will be fully informed of the pros and cons of COLD and PEID before surgery, and given our professional opinion. Ultimately, they are allowed to choose the surgical procedure.

**Fig. 1:**
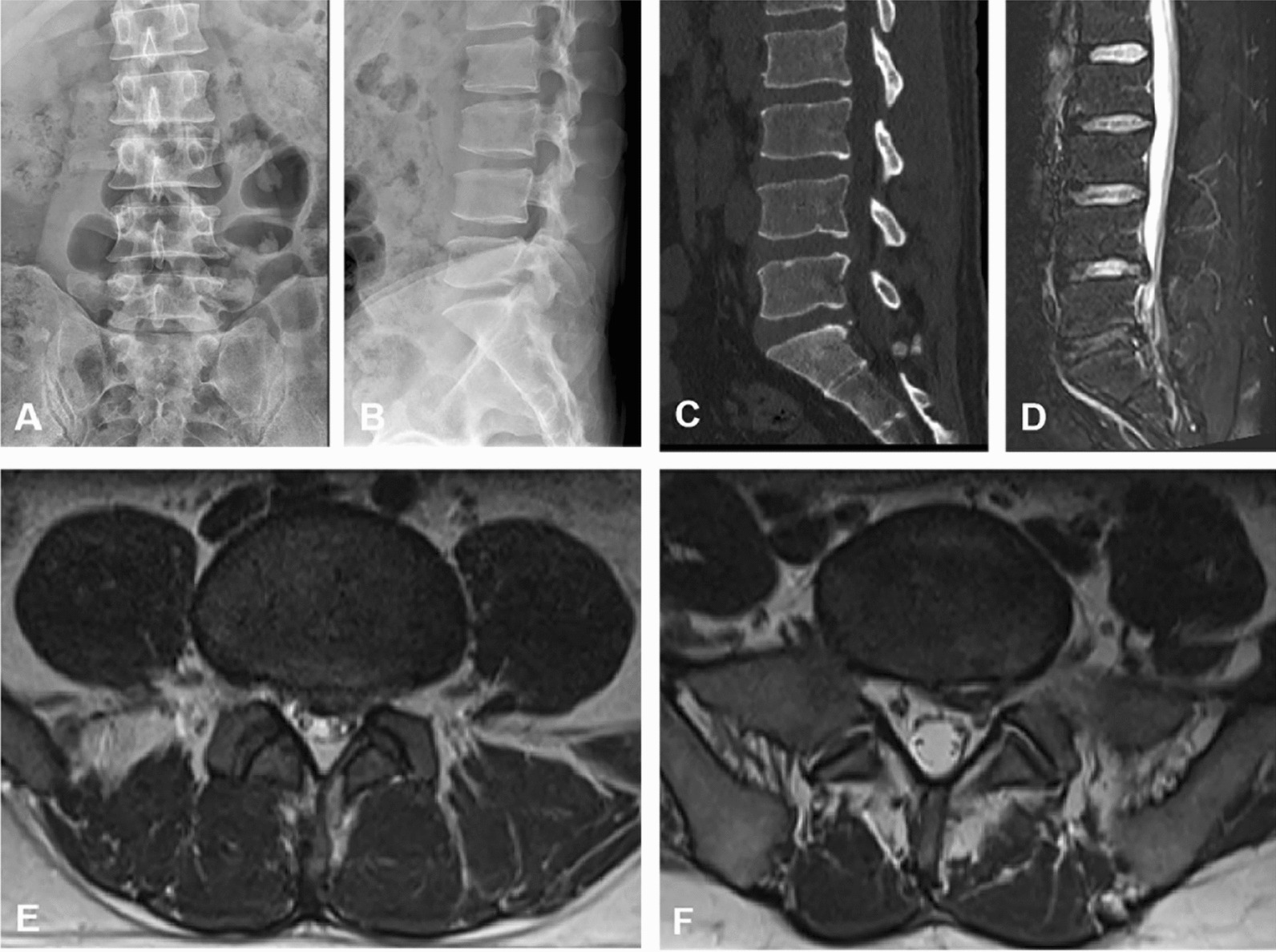
Preoperative radiological examination of a 48-year-old male patient with L4/5 and L5/S1 LDH. **A**–**B**: Frontal and lateral X-ray images. **C**: Sagittal CT image. **D**: Sagittal MRI image. **E**: Cross-sectional MRI image of L4–L5. **F**: Cross-sectional MRI image of L5-S1

The inclusion criteria were as follows: (1) patients aged between 18 and 50 years, (2) those with symptoms of lower back pain and lower limb pain or numbness, (3) those whose magnetic resonance imaging (MRI) and computed tomography (CT) scans confirmed L4/5 and L5/S1 disk herniations with nerve roots compressed (Fig. 1), (4) those with corresponding symptoms caused by the compressed nerve roots, (5) those whose symptoms were not relieved even after 3 months of conservative treatment, (6) those who underwent treatment with COLD or PEID, and (7) those underwent all surgeries by the same surgical team of two experienced surgeons.

The exclusion criteria were as follows: (1) patients who had disk herniations in other levels, (2) those with lumbar spondylolisthesis or other spinal disorders, (3) those with recurrent disk herniations after an open discectomy or PEID, (4) those with cauda equina syndrome, (5) those who were unable to complete the follow-up evaluation, and (6) those with extreme lateral lumbar disk herniation.

### Surgical techniques

#### PEID

**Fig. 2:**
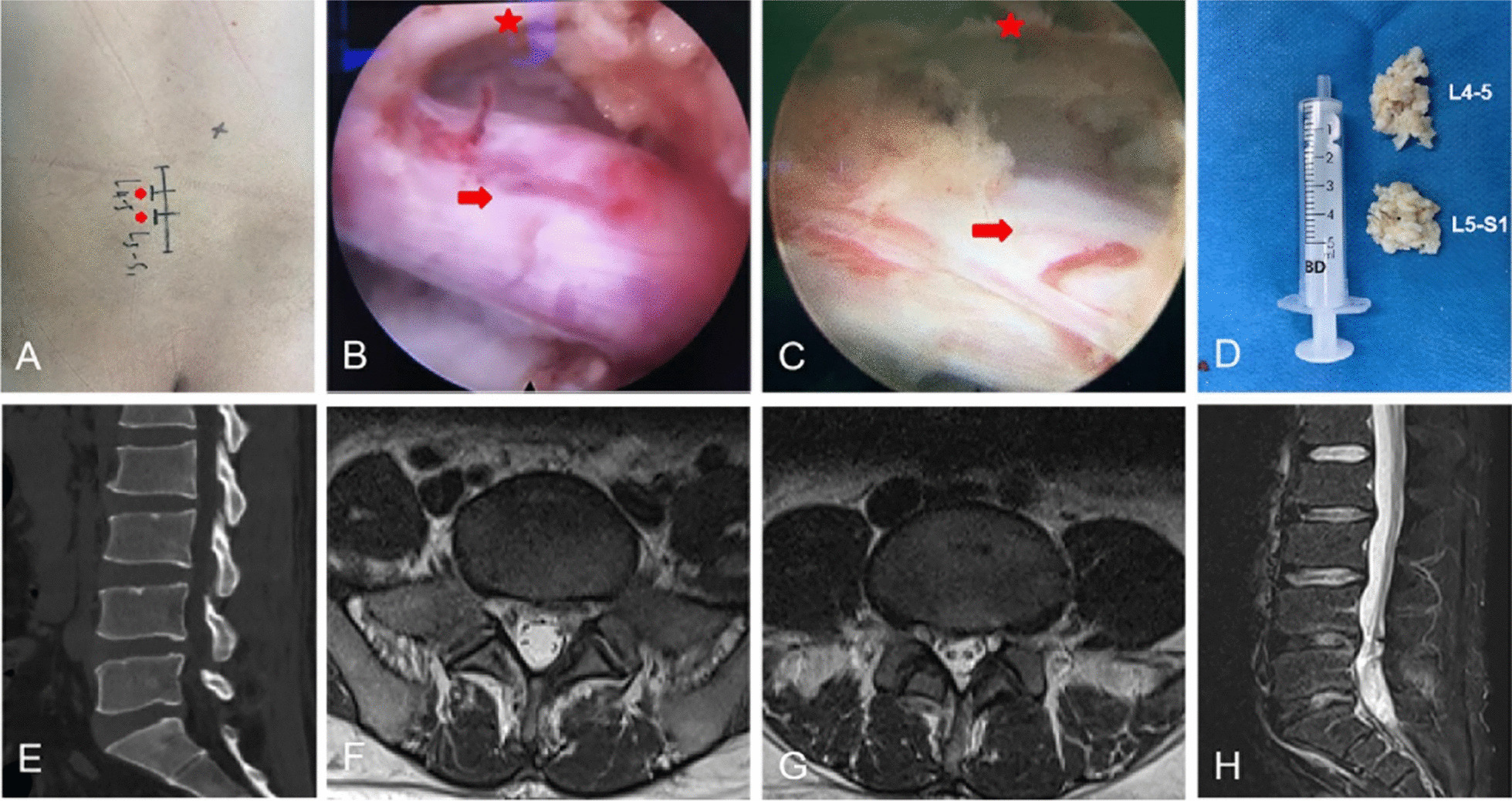
Intraoperative images and postoperative radiological examination. **A**: Preoperative incision planning (red arrow). **B**: Intraoperative view of the interlaminar access with L5 nerve root (arrow) and dural sac (star). **C**: Intraoperative view of the interlaminar access with S1 nerve root (arrow) and dural sac (star). **D**: Disk pulposus. **E**–**H**: CT and MRI at 3 months postoperatively showing good decompression of nerve root and dura

After general anesthesia, patients were placed in the prone position. The operative segment was localized using C-arm. The entry point was chosen at the inferior edge of the superior lamina on the lesion side; an incision of about 5–7 mm was made. We then inserted the spine needle vertically and slightly below the interlaminar center, along the lateral edge of the interlaminar window. After the working cannula was inserted over the dilators, the endoscope was introduced with continuous inflow saline. The ligamentum flavum and epidural fat were carefully removed by different graspers via endoscopic vision. If the laminar space was relatively small, part of the bone in the medial edge of the articular process and the lower edge of the upper lamina were removed. Then, we used the nucleus pulposus forceps to remove the exposed herniated fragment. During operation, the radiofrequency electrode was used intraoperatively to regulate hemostasis and dissect the disk fragments. The working cannula was rotated to further clean the residual disk tissues at the shoulder region. The scope with the cannula was gradually removed under direct visual control. Finally, the incision was closed with a single stitch. Then, another segmental LDH was operated on similarly (Fig. 2).

#### COLD

We performed COLD under direct vision through the posterior interlaminar or translaminar approach. The surgeries were performed under general anesthesia. A 5–7 cm posterior midline incision was made. Next, we performed a lumbar laminectomy and partial laminectomy of the lesser joints along with the removal of the ligamentum flavum, exposing the epidural space and herniated disks compressing the neural tissue. The herniated disk was then exposed and removed, with the nerve root decompressed. Another segment was operated similarly. Complete hemostasis and standard wound closure were performed.

### Clinical assessments

We collected the following preoperative and postoperative clinical data 1 day, 3 months, and final follow-up postoperatively. Preoperative general information included sex, mean follow-up time, age, body mass index, smoking rates, signs, symptoms, and location of LDH. Postoperative data included operative time, intraoperative fluoroscopy time, incision length, postoperative bedtime, and hospital stays. The visual analog scale (VAS) and Oswestry disability index (ODI) were used to assess preoperative symptoms and curative efficacy after surgery. During the hospital stays and follow-up period, the complications, including postoperative dysesthesia and recurrent disk herniation, were counted and addressed. The modified MacNab criteria were used to assess the satisfaction rate for clinical outcomes: "excellent outcome," "good outcome," "fair outcome," and "poor outcome" represent the four different satisfaction levels.

### Statistical analysis

An independent statistician performed statistical analysis with SPSS version 14.0 K (SPSS Inc., Chicago, IL, USA). For the comparison of continuous variables between two groups, the independent two-sample *t* test was applied. Paired* t* test was used to compare the data at different time points in the same group. The Fisher’s exact test was used to compare categorical variables between the two groups. *P* < 0.05 was considered statistically significant.

## Results

### Demographics

The PEID group had 18 male and 7 female patients (mean age = 40.44 ± 8.23 years). The COLD group included 17 male and 8 female patients (mean age = 37.80 ± 9.35 years). The mean follow-up duration was 37.3 months with a minimum of 24 months (range: 24–48 months). No significant difference was observed between the groups regarding mean follow-up time, sex, age, body mass index, smoking rate, symptoms, and physical signs (Table 1).

**Table 1:** General information between the two groups

	PEID	COLD	*t* or *X*^2^ value	*P* value
Number	25	25		
Age	40.44 ± 8.23	37.80 ± 9.35	1.060	0.295
Sex (male/female)	18/7	17/8	0.095	0.758
Mean follow-up time	37.04 ± 7.36	38.16 ± 7.26	− 0.542	0.590
BMI (kg/m^2^)	23.54 ± 3.63	25.11 ± 3.83	− 1.493	0.142
Smoking rate	5/20	7/18	0.439	0.508
Symptoms				
Low back pain	21	22		
Leg pain	24	25		
Signs				
Lasegue test (** +)**	24	23		
Enhanced Lasegue test (** +)**	23	22		
Achilles tendon reflex weakness	19	18		
Location				
L4/L5 (left) & L5/S1 (left)	9	11		
L4/L5 (right) & L5/S1 (right)	13	12		
L4/L5 (left) & L5/S1 (right)	2	1		
L4/L5 (right) & L5/S1 (left)	1	1		

*BMI* body mass index

### Clinical results

In the PEID group, the mean VAS score for back pain was lowered from 5.36 ± 1.25 (preoperatively) to 2.52 ± 1.12 (1 day postoperatively), 2.04 ± 0.98 (3 months postoperatively), and 0.84 ± 0.55 (final follow-up). The mean VAS score for the leg was decreased from 7.68 ± 1.07 (preoperatively) to 3.28 ± 0.98 (1 day postoperatively), 2.24 ± 0.97 (3 months postoperatively), and 0.92 ± 0.40 (final follow-up). Similarly, in the COLD group, the average VAS score for back pain decreased from 5.04 ± 1.27 (preoperatively) to 2.72 ± 0.84 (1 day postoperatively), 2.32 ± 0.80 (3 months postoperatively), and 1.12 ± 0.60 (final follow-up). The mean VAS score for the leg was decreased from 7.80 ± 1.26 (preoperatively) to 3.12 ± 0.88 (1 day postoperatively), 2.32 ± 0.80 (3 months postoperatively), and 1.04 ± 0.61 (final follow-up). In the PEID group, the mean ODI scores preoperatively and at 1 day, 3 months, and final follow-up postoperatively were 57.16 ± 11.40, 24.96 ± 4.13, 17.68 ± 4.27, and 10.48 ± 3.23, respectively. In the COLD group, the mean ODI scores were 56.40 ± 12.80, 26.10 ± 5.77, 17.28 ± 4.90, and 12.16 ± 3.60, respectively. No significant difference was noted in the VAS score and ODI scores between the two groups at 1 day, 3 months, and final follow-up postoperatively. The modified MacNab criteria showed that 23 out of 25 patients in the PEID group and 22 out of 25 patients in the COLD group were excellent or good. No significant difference was noted between the two groups (Table 2).

**Table 2:** Clinical comparison between two groups

	PEID	COLD	*t* or *X*^2^ value	*P* value
VAS back				
Preoperative	5.36 ± 1.25	5.04 ± 1.27	0.895	0.375
1 day	2.52 ± 1.12*	2.72 ± 0.84*	− 0.712	0.480
3 months	2.04 ± 0.98*	2.32 ± 0.80*	− 1.107	0.274
Final follow-up	0.84 ± 0.55*	1.12 ± 0.60*	− 1.715	0.093
VAS leg				
Preoperative	7.68 ± 1.07	7.80 ± 1.26	− 0.363	0.718
1 day	3.28 ± 0.98*	3.12 ± 0.88*	0.607	0.547
3 months	2.24 ± 0.97*	2.32 ± 0.80*	− 0.318	0.752
Final follow-up	0.92 ± 0.40*	1.04 ± 0.61*	− 0.822	0.425
ODI				
Preoperative	57.16 ± 11.40	56.40 ± 12.80	0.449	0.655
1 day	24.96 ± 4.13*	26.10 ± 5.77*	− 1.014	0.315
3 months	17.68 ± 4.27*	17.28 ± 4.90*	0.308	0.759
Final follow-up	10.48v3.23*	12.16 ± 3.60*	− 1.736	0.089
MacNab evaluation				
Excellence	19	17		
Good	4	5		
Fair	1	2		
Poor	1	1		
Excellence/good rate	23/25	22/25	0.200	0.655

*VAS* Visual analog scale; *ODI* Oswestry disability index

*Statistically significant compare with the preoperative, *P* < 0.05

### Surgical results

Among the parameters, the operation time was longer in the PEID group compared to the COLD group (96.60 ± 20.69 min vs. 77.88 ± 17.74 min, *P* < 0.05) The intraoperative fluoroscopy time was more in the PEID group than in the COLD group (3.56 ± 0.58 times vs. 1.24 ± 0.44 times). The incision length was 1.28 ± 0.11 cm in the PEID group and 6.73 ± 0.88 cm in the COLD group. The postoperative bedtime was 8.64 ± 1.08 h in the PEID group and 20.64 ± 2.78 h in the COLD group. The hospital stays were 2.16 ± 0.47 days in the PEID group and 4.04 ± 0.89 days in the COLD group. In terms of incision length, postoperative bedtime, and hospital stays, the PEID group was better than the COLD group (*P* < 0.05, Table 3).

**Table 3:** Comparison of intraoperative outcomes between the two groups

	PEID	COLD	t value	P value
Operation time (min)	96.60 ± 20.69	77.88 ± 17.74	6.177	< 0.05
Intraoperative fluoroscopy time	3.56 ± 0.58	1.24 ± 0.44	15.934	< 0.05
Incision length (cm)	1.28 ± 0.11	6.73 ± 0.88	− 30.878	< 0.05
Postoperative bed time (h)	8.64 ± 1.08	20.64 ± 2.78	− 20.116	< 0.05
Hospital stays (d)	2.16 ± 0.47	4.04 ± 0.89	− 9.338	< 0.05

*min* minutes; *cm* centimeters; *h* hours; *d* days

### Complications

Complications occurred in two of the PEID group and three of the COLD group. One patient in the PEID and COLD groups developed postoperative leg numbness, which was relieved within 1 month postoperatively by conservative treatment such as swelling reduction, pain relief, and bed rest. This might have been caused by postoperative dysesthesia due to the dorsal root ganglion edema caused by mechanical stretch or damage [11]. One patient (4.0%) suffered recurrent L4/L5 disk herniation at 13 months in the PEID group, and the pain was relieved after conservative treatment. Two patients (8.0%) in the COLD group suffered from recurrent disk herniation at 8 and 17 months, respectively. One patient suffered from L4/L5 disk herniation and underwent conservative treatment, and the other one suffered from L5/S1 disk herniation and was treated with revision surgery. No other serious complications such as dural tear, urinary retention, poor wound healing, and intraoperative nerve root injury were observed (Table 4).

**Table 4:** Complications between the two groups

	PEID	COLD	*X*^2^ value	*P* value
Total complications	2(8.0%)	3(12.0%)	0.000	1.000
Intraoperative nerve root injury	0	0		
Leg pain/numbness	1(4.0%)	1(4.0%)	0.000	1.000
Dural tear	0	0		
Urinary retention	0	0		
Poor wound healing	0	0		
Recurrence cases	1(4.0%)	2(8.0%)	0.000	1.000

## Discussion

COLD and PELD are effective in relieving pain or numbness in the lower back and legs caused by nerve compression for the treatment of single-segmental LDH [9]. COLD is still the standard procedure for the treatment of LDH, with few complications and satisfactory outcomes. It has significant advantages in the treatment of complex LDH, such as larger LDH, extreme lateral LDH, and LDH accompanied by spinal stenosis [5, 6, 12]. However, with the development of microscopic techniques and the improvement of surgical skills, because of its advantages of less bleeding and soft tissue damage, maintenance of spinal stability, shorter hospital stays, and enhanced recovery after surgery, PELD is slowly replacing COLD as the gold standard for LDH [13]. At the same time, some surgeons find that PELD can also manage part complex LDH such as larger LDH [14–16]. Double-segmental LDH is more complex to diagnose and difficult to treat than single-segmental LDH because the clinical symptoms do not match the imaging presentation. Microdiscectomy has tremendous advantages for the treatment of single-segment LDH, whereas the treatment of double-segmental LDH at L4/5 and L5/S1 remains controversial. In PEID, the disk is reached through the lamina and ligamentum flavum, a surgical approach that is very similar to COLD. Our study compared the clinical outcomes of PEID and COLD in the treatment of double-segmental LDH and showed the efficiency of PEID in dealing with this situation. A clear diagnosis is important for double-segmental LDH. Misdiagnosis leads to unsatisfactory clinical outcomes, whereas excessive surgical treatment causes unnecessary lesions. In our study, we referred to the segments that cause symptoms as the responsible segments, which were visible in CT or MRI with clear disk herniation points, definitely compressed nerve roots, and obvious nerve root edema around the protrusion. The affected nerve roots that are diagnosed with radiology must be consistent with those identified by signs and symptoms. Briefly, “matching symptoms, signs, and images” is the principle to determine the responsible segment.

Few studies have reported the clinical outcome of double-segmental PEID. Wu et al. reported two segmental PEIDs in the treatment of far-migrated disk herniation and obtained satisfactory outcomes [17]. PEID and COLD achieved satisfactory clinical outcomes for single-segmental LDH [18–22]. According to our results, no significant difference was found in the clinical outcomes or satisfactory rates between the two approaches. In our study, the PEID group was able to better expose and remove the disks compressing the nerve roots using an endoscope, with lesser removal of the paraspinal soft tissue and bone tissue in the region of interarticularis and facets. COLD required stripping of the paravertebral muscles and biting off part of the articular eminence to fully expose the surgical field, resulting in the weakening of the local muscles, more scarring, and loss of the elasticity of normal muscle tissue [23]. Therefore, we recommend that patients undergoing COLD should appropriately extend their postoperative bedtime and hospital stay. The PEID group had faster recovery and shorter hospital stays than the COLD group. Conversely, PEID showed advantages such as paraspinal structures preservation, lesser blood loss, and lower risk of epidural scar formation and iatrogenic instability, consistent with previous studies [24–26]. In terms of the operation time, Song et al. [27] showed that PEID required a shorter operative time than open discectomy. Open discectomy requires layer-by-layer opening and closure, frequent laminectomies, and drainage tube insertion, thus leading to a significant increase in the operative time in the treatment of L5/S1 LDH. Conversely, when dealing with L4/L5 and L5/S1 double-segment LDH, we found that the operating time in the COLD group was significantly shorter than that in the PEID group. Double-segment PEID increased the intraoperative fluoroscopy times; moreover, when dealing with an L4/5 LDH, PEID required resection of the medial portion of the superior articular bulge and the lower edge of the L4 lamina due to the narrow lumbar vertebral lamina space [28].

All patients in the PEID group were treated using a double-incision approach. The double incision provided a smoother puncture, reduced operational difficulties, and led to better exposure of the compressed nerve root in the event of herniation at different sites. Although PEID is a minimally invasive procedure, COLD is a simpler approach for beginners to master. Therefore, we advise that new surgeons should perform COLD before PEID. In conclusion, our study followed up the advantages and disadvantages of COLD or PEID for the treatment of patients with LDH at L4/5 and L5/S1 double-segmental LDH, which will help spine surgeons to fully understand the characteristics of the two procedures so that they can make a more appropriate choice for their patients.

### Limitations

There are several limitations to our study. First, the sample sizes of the two groups were relatively small. Thus, more patients will be covered in future study. Second, the study design was retrospective. Therefore, prospective randomized controlled trials are needed in the future, which would better support our conclusions and would avoid sample size reduction due to loss of follow-up information.

## Conclusion

PEID and COLD have similar therapeutic effects and safety for L4/5 and L5/S1 double-segmental LDH. The complication and recurrence rates of PEID are comparable to those of COLD. According to our findings, PEID exhibits more advantage in surgical trauma control and enhanced recovery after surgery compared to COLD.

## Data Availability

The datasets used and/or analyzed during the current study are available from the corresponding author on request.

## Abbreviations

PELD: Percutaneous endoscopic lumbar discectomy
PEID: Percutaneous endoscopic interlaminar discectomy
COLD: Conventional open lumbar discectomy
LDH: Lumbar disk herniation
VAS: Visual analog scale
BMI: Body mass index
ODI: Oswestry disability index
CT: Computed tomography
MRI: Magnetic resonance image
